# Genome-Wide Comparative Analysis of 20 Miniature Inverted-Repeat Transposable Element Families in *Brassica rapa* and *B. oleracea*

**DOI:** 10.1371/journal.pone.0094499

**Published:** 2014-04-18

**Authors:** Perumal Sampath, Jayakodi Murukarthick, Nur Kholilatul Izzah, Jonghoon Lee, Hong-Il Choi, Kenta Shirasawa, Beom-Soon Choi, Shengyi Liu, Ill-Sup Nou, Tae-Jin Yang

**Affiliations:** 1 Department of Plant Science, Plant Genomics and Breeding Institute, and Research Institute for Agriculture and Life Sciences, College of Agriculture and Life Sciences, Seoul National University, Seoul, Republic of Korea; 2 Department of Plant Genome Research, Kazusa DNA Research Institute, Chiba, Japan; 3 National Instrumentation Center for Environmental Management, College of Agriculture and Life Sciences, Seoul National University, Seoul, Republic of Korea; 4 The Key Laboratory of Oil Crops Biology and Genetic Breeding, the Ministry of Agriculture, Oil Crops Research Institute, the Chinese Academy of Agricultural Sciences, Wuhan, China; 5 Department of Horticulture, Sunchon National University, Suncheon, Republic of Korea; Ben-Gurion University, Israel

## Abstract

Miniature inverted-repeat transposable elements (MITEs) are ubiquitous, non-autonomous class II transposable elements. Here, we conducted genome-wide comparative analysis of 20 MITE families in *B. rapa*, *B. oleracea*, and *Arabidopsis thaliana*. A total of 5894 and 6026 MITE members belonging to the 20 families were found in the whole genome pseudo-chromosome sequences of *B. rapa* and *B. oleracea*, respectively. Meanwhile, only four of the 20 families, comprising 573 members, were identified in the *Arabidopsis* genome, indicating that most of the families were activated in the *Brassica* genus after divergence from *Arabidopsis*. Copy numbers varied from 4 to 1459 for each MITE family, and there was up to 6-fold variation between *B. rapa* and *B. oleracea*. In particular, analysis of intact members showed that whereas eleven families were present in similar copy numbers in *B. rapa* and *B. oleracea*, nine families showed copy number variation ranging from 2- to 16-fold. Four of those families (BraSto-3, BraTo-3, 4, 5) were more abundant in *B. rapa*, and the other five (BraSto-1, BraSto-4, BraTo-1, 7 and BraHAT-1) were more abundant in *B. oleracea*. Overall, 54% and 51% of the MITEs resided in or within 2 kb of a gene in the *B. rapa* and *B. oleracea* genomes, respectively. Notably, 92 MITEs were found within the CDS of annotated genes, suggesting that MITEs might play roles in diversification of genes in the recently triplicated *Brassica* genome. MITE insertion polymorphism (MIP) analysis of 289 MITE members showed that 52% and 23% were polymorphic at the inter- and intra-species levels, respectively, indicating that there has been recent MITE activity in the *Brassica* genome. These recently activated MITE families with abundant MIP will provide useful resources for molecular breeding and identification of novel functional genes arising from MITE insertion.

## Introduction

The Brassicaceae is one of the largest and most important plant families, with 338 genera and about 3709 species including many economically significant vegetable crops, oil seed plants, condiments and fodder crops [1], [2]. The genomic relationship between the six interrelated cultivated *Brassica* species, including three diploids [*Brassica rapa* (2*n* = 2x = 20, AA genome, 529 Mb genome size), *B. nigra* (2*n* = 2x = 16, BB, 632 Mb), *B. oleracea* (2*n* = 2x = 18, CC, 696 Mb)] and three amphidiploid derivatives [*B. juncea* (2*n* = 4x = 36, AABB, 1068 Mb), *B. napus* (2*n* = 4x = 38, AACC, 1132 Mb), and *B. carinata* (2*n* = 4x = 34, BBCC, 1284 Mb)] has been summarized as the triangle of U [3], [4]. Due to its wide distribution and the differences in polyploidy of its members, the Brassicaceae provides an excellent system in which to study polyploidization-mediated evolution of plants [5]. Comparative studies with the diploid model plant *A. thaliana* (*2n* = 2x = 10, 125 Mb) have confirmed approximately 16-fold genome size variation in the Brassicaceae [6], [7]. Studies have also revealed that *B. rapa* and its close relative *B. oleracea* evolved as whole genome triplication derivatives after their split with *A. thaliana* 13–17 million years ago (MYA) [7]. Sub- or neo-functionalization is one of the important driving forces for maintenance of genome integrity in genomes subjected to whole-genome duplication [8]–[10]. Genome-wide comparative analysis has revealed that whole-genome duplication is the major factor responsible for the increase of genome size in *Brassica*. In addition to the polyploidization events, accumulation and amplification of transposable elements (TEs) contribute to increased genome size in *Brassica* species [11], [12].

TEs are fundamental agents for genome size enlargement and evolution [13]–[17], and are classified as class I (retrotransposons) or class II (DNA transposons) mobile genetic elements based on their transposition mechanism. TEs that contain their own coding sequences for transposition are called autonomous TEs. Conversely, TEs with defective or no coding sequences are referred to as non-autonomous [13], [16], [18]. Miniature inverted-repeat transposable elements (MITEs), class II-type TEs, are relatively small in size (<800 bp) and share conserved structural characteristics, such as terminal inverted repeats (TIRs) and target site duplication (TSD). MITEs are A+T-rich (>50–65%) [19], and preferentially insert into inter-genic, near-genic or intronic regions but usually avoid exonic regions [20]. There are two major superfamilies of MITEs, namely *Stowaway* (with TA as the TSD) and *Tourist* (with TAA as the TSD), and several other minor families such as *hAT* (5, 6, or 8 bp TSDs), *Mutator* (9–10 bp TSDs), and *En/Spm* (3 bp TSDs) [21]–[24]. In plants, MITEs are present in tens of thousands of copies throughout the entire genome and influence genomic diversity and differentiation [14], [25]. Indeed, MITEs can occupy a major fraction of plant genomes, up to 10% in rice, 8% in *Medicago*, 4% in *B. rapa* and 0.71% in *A. thaliana* [26].

MITEs are active and important players in gene and genome evolution [27], [28]. Due to their close association with genes or specific genic regions such as introns, exons, untranslated regions (UTRs) and promoters, MITEs can alter or disturb gene structure, expression, and/or function [14], [18], [29]–[31]. MITEs have been reported to be involved in the alteration of triplicated genes in *B. rapa* and up- or down-regulated gene expression [9], [20]. MicroRNAs are small non-coding RNAs (21–24 nt) that regulate specific target genes or transposable elements at the transcriptional and post-transcriptional level. Recent studies suggest that 20% of the known miRNAs in the human genome originated from TEs [32]. MicroRNAs derived from MITEs through stem-loop structures play an important role in silencing TEs [33]–[36]. Because of the well-defined MITE boundaries including the TIRs and TSD, *de novo* identification of MITEs has become possible using tools like MUST, RSBP, MAK, MITE-Hunter and MITE Digger [23], [37]–[40]. In addition, previously annotated information for MITE homologs has enabled homology-based MITE identification with tools like CENSOR at the Repbase database and RepeatMasker [23], [37], [38]. MITEs can be an excellent resource for the development of DNA markers for genomics and evolutionary studies because most are stably inherited and present in high copy numbers [20], [41]–[44].

TEs are one of the major factors contributing to genome size in the highly duplicated *Brassica* genome [11] and are thought to occupy 39.5% and 38.8% of the genome in *B. rapa* and *B. oleracea*, respectively [12], [45], for which whole-genome sequence information is now publicly available [12], [46]. Recent genome-wide characterization of MITEs using various *in silico* tools has revealed 174 MITE families in *B. rapa* including 90 *hAT*, 56 *Tourist* and 16 *Stowaway*, 11 *Mutator* and 1 *CACTA* families. A total of 45821 MITE members occupy >11 Mb (4.08%) of the *B. rapa* genome [26]. However, there have been only a few comparative genomics studies on transposable elements, especially MITEs, and their practical application in breeding and evolutionary studies of the *Brassica* genome [11], [20], [47], [48]. Among these is our previous characterization of a high-copy *Stowaway* MITE family (BraMi-1), which highlighted the utility of MITEs as molecular markers and the importance of MITEs in the *Brassica* genome for genomic and breeding purposes [20]. Now, we have conducted genome-wide comparative analysis of 20 MITE families in *B. rapa* and *B. oleracea* to provide a basis for understanding MITE dynamics in the *Brassica* genome. Our analysis explores all members of 20 MITE families, including three previously unknown families, and their distribution in *B. rapa* and *B. oleracea*. Furthermore, the potential utility of MITEs as molecular markers for genomics is demonstrated.

## Materials and Methods

### Identification and characterization of MITEs in the *B. rapa* and *B. oleracea* genomes

The whole genome pseudo-chromosome sequences with unanchored scaffolds for *B. rapa* (283 Mb) *Version 1.2* and pseudo-chromosome sequences of *B. oleracea* (385 Mb) *Version 1.0*, with their gene annotation information, were obtained from the public databases BRAD [49] and BolBase [46], respectively. The MITE-Hunter program [38] was used for identification of the MITEs in the *B. rapa* and *B. oleracea* genomes with default parameters. The putative MITE sequences generated by MITE-Hunter were characterized using the BLAST 2 sequences tool from NCBI (http://blast.ncbi.nlm.nih.gov/Blast.cgi) and the EMBOSS application ‘einverted’ (http://emboss.bioinformatics.nl/cgi-bin/emboss/einverted) to identify the TIR and TSD structures. The expected hairpin structures were estimated using one representative intact member of the each MITE family using mfold [50]. The identified MITEs were searched against Repbase, RepeatMasker and P-MITE database [26], [51], [52] to find the homologous MITEs from plant species. MITE families were searched for MITE-derived miRNA at miRbase version 19 using an E-value of <e-10 against the Brassicaceae species [53].

### MITE members in the *B. rapa a*nd *B. oleracea* genomes and their phylogeny

To retrieve the MITE members and study the distribution pattern of each MITE family, the consensus sequences for the MITE families were used as queries in BLASTN searches against the pseudo-chromosome sequences of *B. rapa* and *B. oleracea*. The members of each MITE family were extracted from each genome using an E-value of <e-5. The duplicate hits from the same physical positions were removed by manual analysis in order to count exact copy numbers. MITE insertion positions of family members from *B. rapa* (Br-members) and *B. oleracea* (Bo-members) were characterized using gene annotation information by a custom Perl script. MITE members with ≥80% alignment length and ≥80% identity, hereafter designated as 80∶80 coverage, were considered to be intact candidate member for each MITE family according to 80∶80∶80 rule. The third 80 of this rule denotes that the element has ≥80 bp sequence similarity to a TE family and can therefore be considered a member of that TE family; it is also possible for an element with <80 bp sequence similarity to be considered a member of the TE family upon in-depth analysis [16]. Phylogenetic analysis was conducted using 20% of the intact MITE members for each family, because there were too many members in certain families. Based on a ClustalW sequence alignment, a phylogenetic tree was generated using the neighbor-joining method with 500 replications in MEGA5 [54].

### MITE insertion polymorphism analysis

The presence or absence of a MITE in a particular locus can produce polymorphism between accessions, which can be analyzed using MITE insertion polymorphism (MIP) analysis [44]. MIP was surveyed using 346 MITE-flanking primer pairs against eight accessions including two *Brassica* diploid species *B. rapa* (A genome), *B. oleracea* (C genome) and a corresponding tetraploid species *B. napus* (AC genome), as well as *A. thaliana*. The details for the plant materials, e.g., their ploidy and subgroup of the *Brassica* genome, were the same as in the previous MIP analysis [20]. Total genomic DNA was extracted from fresh leaves using the modified CTAB method [55]. Primer information and expected product size, generated by the Primer3 program [56], are listed in Table S1 in File S1. PCR mixtures (20 µL total) consisted of 10 ng DNA, 1× PCR buffer, 0.2 µM each primer, 2.5 µM dNTPs, and 1 unit *Taq* DNA polymerase (VIVAGEN, Korea). PCR was carried out as 5 min at 94°C, 35 cycles of 95°C for 30 sec, 56°C–62°C (dependent on the primers) for 30 sec, and 72°C for 1 min, with a final 5-min extension at 72°C, using a MG96G thermo cycler (LongGene Scientific Instruments, China). The PCR products were separated on 2% agarose gels, and the gels were stained with ethidium bromide and visualized on a UV trans-illuminator.

## Results

### Identification of 20 MITE families in the *B. rapa* and *B. oleracea* genomes

**Figure 1:**
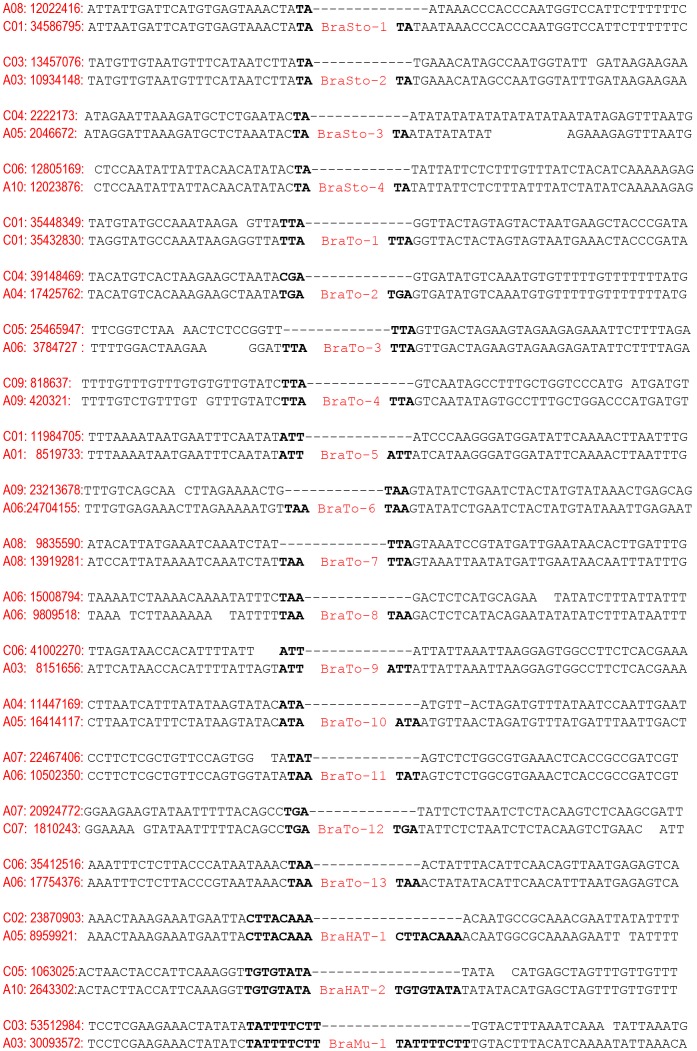
Identification of related empty sites by sequence comparison of MITE flanking regions with corresponding regions from *B. rapa* (A genome) or *B. oleracea* (C genome) homologs. The chromosome numbers and start positions of the sequences are indicated at the beginning of the sequence. TSD sequences are shown in bold.

Genome-wide analysis of MITEs using the MITE-Hunter program yielded 145 and 175 putative MITEs from *B. rapa* and *B. oleracea*, respectively. These 320 putative MITEs were characterized for the basic structural characteristics of MITEs including TIRs and TSD using the BLAST 2 sequences, *einverted* application with manual annotation, and we thereby identified 20 MITE families including the two previously known MITE families BrMi-1 (herein termed BraSto-1, as described below) and BraMi-1 (herein termed BraSto-2) [20], [47], [48]. Each family was characterized by short length, ranging from 160 to 556 bp, and had TIR sequences of between 16 and 85 bp. The identified MITE families had >50% A+T content, as is usual for MITEs, with one exception (BraTo-9, 39%). Relative empty site analysis showed that the TSD sequences for the families were different, being 2, 3, 8 and 10 bp (Figure 1). Based on their TSD, the MITE families were classified into one of four superfamilies, *Stowaway*, *Tourist*, *hAT* and *Mutator*. We named the *Stowaway* families as *Brassica Stowaway* (BraSto) 1–4, the *Tourist* families as *Brassica Tourist* (BraTo) 1–13, the *hAT* families as *Brassica hAT* (BraHAT) 1–2 and the *Mutator* family as *Brassica Mutator* (BraMu) 1 (Table S2 in File S1). Homology-based repeat analysis using the CENSOR program in Repbase [51] showed that five families, BraSto-3, BraSto-4, BraTo-2, BraTo-7, and BraTo-8, had sequence similarity to previously reported MITE families. Among them, BraSto-3 and BraTo-2 shared over than 75% sequence similarity with MITEs in other plant families such as *METMITE* in Barrel Clover (*Medicago truncatula*) and *HARB-1N1_Stu* in potato (*Solanum tuberosum*). BraSto-4, BraTo-7, and BraTo-8, showed the highest sequence similarity to *ATPOGO* (80%), *ATTIRX-1B* (72%) and *ATTIRX-1C* (75%) in *A. thaliana*. Subsequent similarity searches against the recently developed plant MITE (P-MITE) database [26] revealed that 3 out of the 20 identified families (BraSto-4, BraTo-11 and BraTo-13) were not represented in that database (Table S2 in File S1).

### Characterization of 20 MITE families in *B. rapa* and *B. oleracea*

Representative sequences of the 20 MITE families were used for retrieving all family members. Overall 5894 (Br-members) and 6026 (Bo-members) MITEs were identified and retrieved from the *B. rapa* and *B. oleracea* genomes, respectively. Of those, 1645 (28%) Br-members and 1604 (27%) Bo-members were relatively intact, maintaining 80∶80 coverage (as described in the Materials and Methods) with E-values of <e-5. However, only 573 members were identified in the *A. thaliana* genome, of which 122 (21%) were intact, belonging to four homologous MITE families (BraSto-2, 4 and BraTo-10, 11) (Tables S2, S3 and S4 in File S1). BraSto-4 was present in the highest copy numbers, with 1369 and 1459, whereas BraSto-3 had the fewest copies, with four and five, in *B. rapa* and *B. oleracea*, respectively.

**Figure 2:**
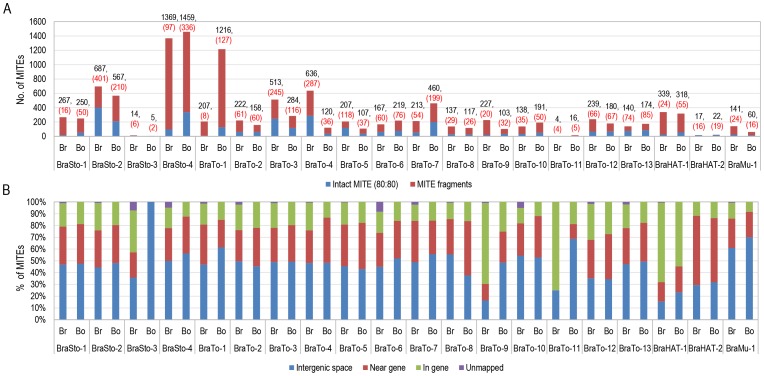
Distribution of 20 MITE families in the *B. rapa* and *B. oleracea* genomes. (A) Total number of members of each of the 20 MITE families in the *B. rapa* and *B. oleracea* genomes. The total numbers of members and intact members (in parentheses) of the corresponding MITE family are given above each bar. (B) Distribution of total MITE family members in various types of genomic sequence in the *B. rapa* and *B. oleracea* genomes (relative percentage). Br and Bo indicate analysis in the *B. rapa* and *B. oleracea* genome, respectively.

Thirteen MITE families were similar in copy numbers between the *B. rapa* and *B. oleracea* genomes, but seven MITE families (BraSto-3, BraTo-1, 4, 7, 9, 11 and BraMu-1) showed 2- to 6-fold (BraTo-1) differences in total copy numbers (Table S2 in File S1). In particular, BraTo-1 was present as only about 207 copies in the *B. rapa* genome, whereas the *B. oleracea* genome contained 1216 copies. Furthermore, high copy number variation between the *B. rapa* and *B. oleracea* genomes was observed for the intact members of nine MITE families. For four families (BraSto-3 and BraTo-3, 4, 5), 2- to 8-fold more intact copies were found in *B. rapa* than in *B. oleracea*. Conversely, five families (BraSto-1, BraSto-4, BraTo-1, 7 and BraHAT-1) were 2- to 16-fold richer in intact copies in *B. oleracea* than in *B. rapa*. For instance, the BraTo-4 and BraSto-4 families showed 7- and 15-fold higher copy numbers in *B. rapa* than in *B. oleracea*. Notably, BraSto-4 had only 97 intact members out of a total of 1369 (7%) homologs in the *B. rapa* genome, whereas 336 out of 1459 (23%) members were intact in *B. oleracea* (Figure 2a, Table S2 in File S1).

Br- and Bo-members occupied approximately 0.38% (0.93 Mb of 283 Mb) and 0.33% (1.05 Mb of 385 Mb) of the available the *B. rapa* and *B. oleracea* whole genome pseudo-chromosome sequences. Secondary structure analysis using one representative intact member of each of the 20 MITE families revealed unique characteristic loops, which might be needed for their transposition (Figure S1 in File S1) [14], [28]. Homology search analysis against an miRNA database (miRBase) revealed that 10 MITEs shared homology with 10 miRNAs reported in *A. thaliana*. MITE-derived miRNAs were identified in various positions of the MITEs, from terminal to internal regions, with some mismatches (up to 7 bases) (Table S5 in File S1).

**Figure 3:**
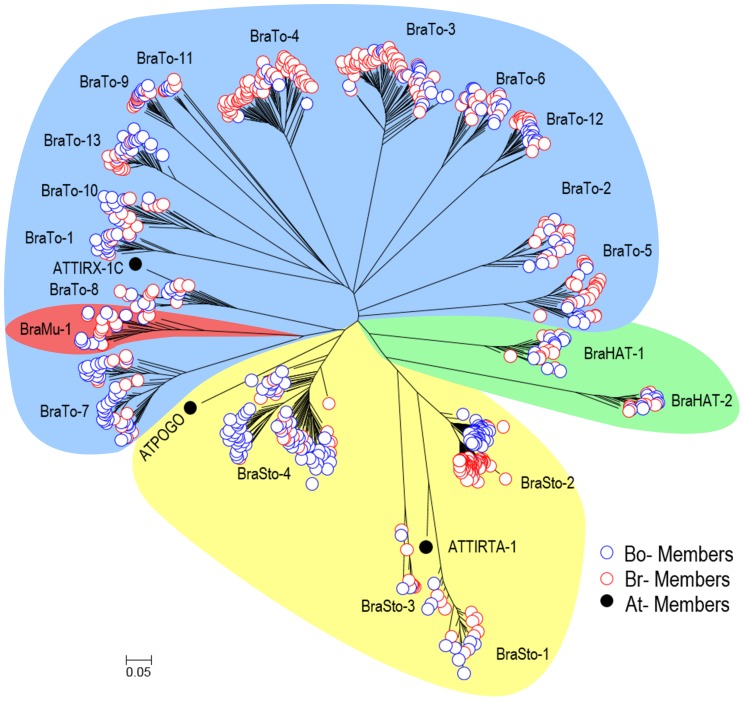
Phylogenetic analysis of candidate MITE families from *B. rapa* and *B. oleracea*. The analysis was performed using 20% of the identified MITE family members having 80∶80 coverage. The 20 MITE families were separated in to different clades by unrooted phylogenetic analysis. The families belonging to the four MITE superfamilies, *Stowaway*, *Tourist*, *hAT* and *Mutator*, are indicated with yellow, blue, green and orange backgrounds, respectively.

Multiple sequence alignment showed overall sequence conservation levels of 66–90% similarity between members in the same MITE families. The TIRs were especially well conserved, showing >90% sequence similarity. Phylogenetic analysis with a representative 20% of intact MITE members (showing 80∶80 coverage), including 367 and 339 members from *B. rapa* and *B. oleracea*, respectively, showed low sequence variation between some family members, while different MITE family members showed clear separation. Br- and Bo-members belonging to the same family were grouped into the same clusters (Figure 3).

### Distribution of MITE family members in the *B. rapa* and *B. oleracea* genomes

**Figure 4:**
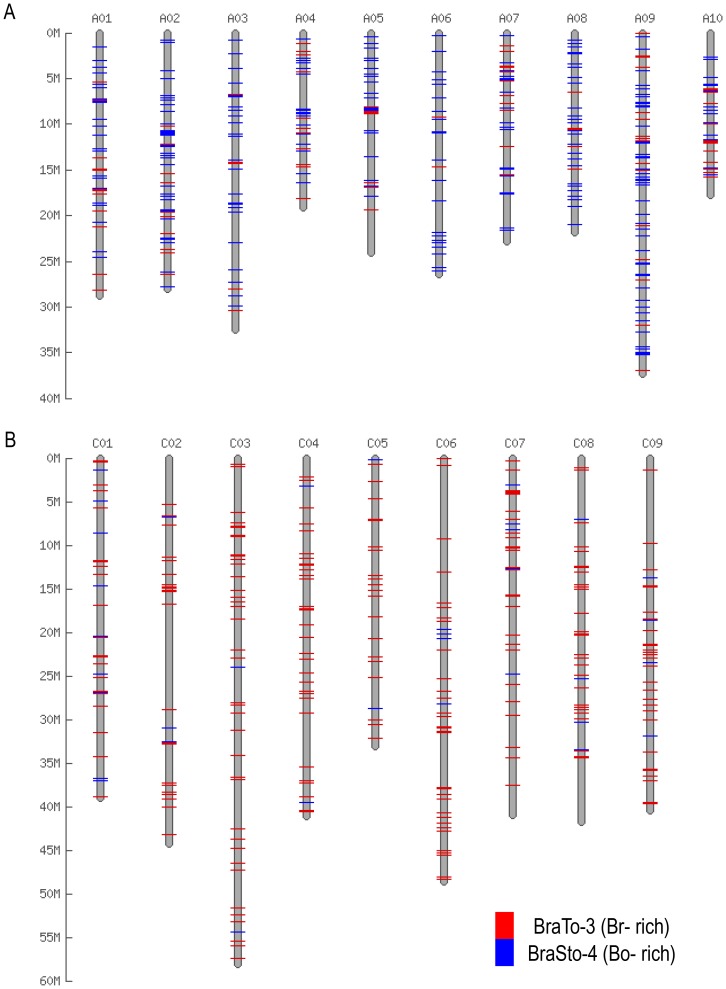
Differential distribution of MITE family members in *B. rapa* and *B. oleracea*. MITE families with intact members were used for *in silico* map construction on the 256 Mb *B. rapa* (A) and the 385 Mb *B. oleracea* (B) pseudo-chromosome sequences based on the physical positions. The physical position information for the MITE families of *B. rapa* and *B. oleracea* are listed in Table S3 and S4, respectively.

The 5894 Br-members and 6026 Bo-members are distributed throughout the chromosomes of the *B. rapa* and *B. oleracea* genome, respectively. Correspondingly, the 573 members from *A. thaliana* were distributed throughout the genome. The 1645 Br-members and 1604 Bo-members that were relatively intact (80∶80 coverage) showed similar distribution patterns to those of all members (Figure 4; Figure S2 in File S1). The MITE insertion positions were characterized based on the whole-genome annotation of *B. rapa* and *B. oleracea*. Among the 5894 Br-members, 5761 were successfully mapped on *B. rapa* pseudo-chromosome sequences while the other 133 members were identified on unallocated scaffold sequences (Figure 2b: Table S6 in File S1). Out of the 5761 Br-members, 2641 (46%), 1675 (28.4%) and 1445 (24.5%) were positioned in intergenic spaces, near genic regions and in genic regions, respectively. Among the 1445 members in the genic regions, 89 (1.5%) and 692 (11.7%) elements were present in the coding sequences (CDSs) and intronic regions, respectively (Figure 2b, Tables S3 and S6 in File S1). We found that 54% of MITEs were present within 2 kb of genic regions, and 664 (11.5%) members resided within the 500 bp up- and down-stream of the start and stop codons (designated as the 5′- and 3′-UTRs), respectively (Table S6 in File S1). Similarly, of the 6026 Bo-members, 49.1% (2958), 31.6% (1906) and 1162 (19.3%) were found in intergenic spaces, near genic regions and in genic regions, respectively. Among the 1162 elements in genic regions, 412 (6.8%), 380 (6.3%), and 367 (6.1%) were in introns, 5′-UTRs and 3′-UTRs, respectively (Figure 2b, Tables S4 and S6 in File S1).

### BraTo-9 MITE family members preferentially reside in genic regions in *B. rapa*

**Figure 5:**
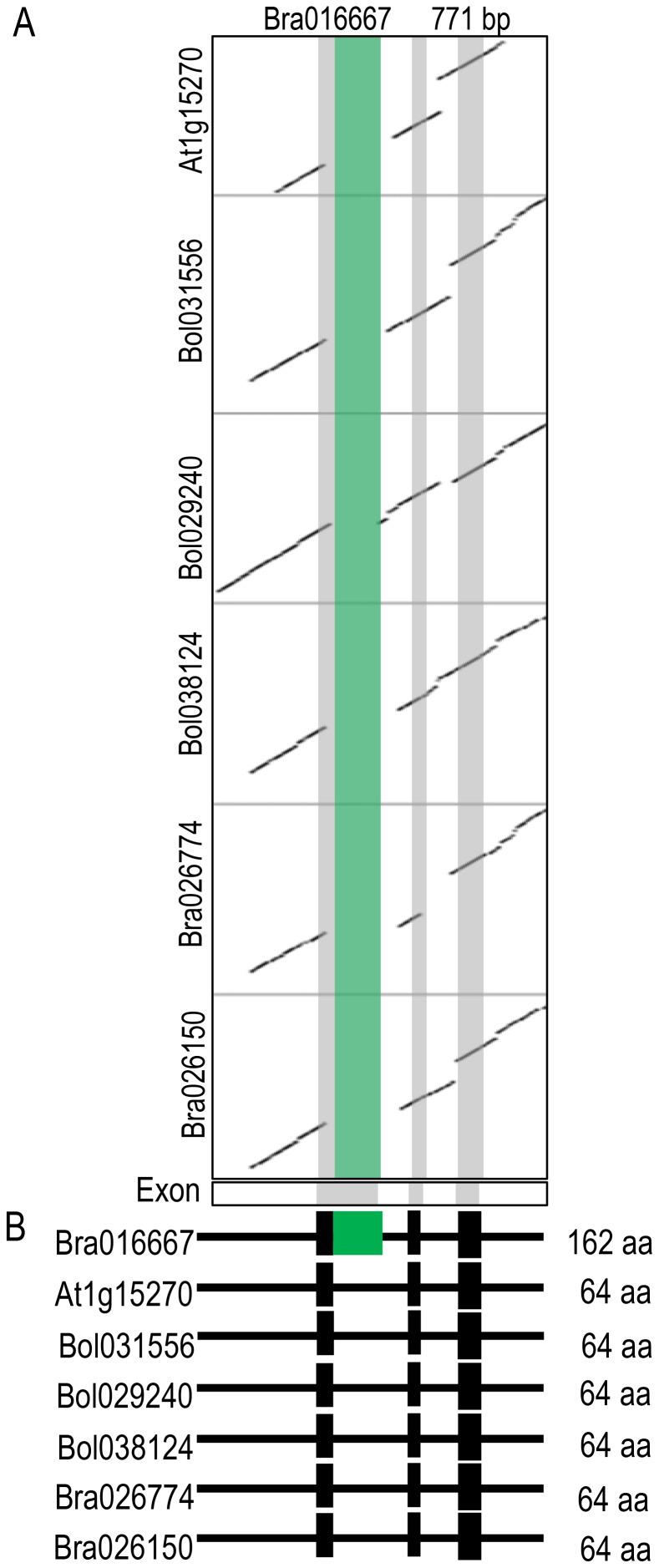
MITE insertion introduced a new exon into the Bra016667 gene of *B. rapa*. (A) Dot-plot comparison of genomic regions of BraTo-9-inserted gene Bra016667 with its paralogs Bra026774, Bra026150 and orthologs (At1g15270, *TRANSLATION MACHINERY ASSOCIATED7*) and Bol038124, Bol029240 and Bol031556 from *B. rapa*, *A. thaliana*, and *B. oleracea*, respectively. (B) Comparison of exon arrays of the genes shown in (A). The MITE based neo-exon is indicated as a green bar. The exon arrays were determined based on similarity to the ortholog At1g15270.

Unlike the members of the other 19 MITE families, BraTo-9 members, including >66% (150/225) of the Br-members, were preferentially accumulated in exons of many genes in *B. rapa* but not in *B. oleracea*. Partial sequences of BraTo-9 elements were expressed as chimeric forms in 29 genes of *B. rapa* (Table 1). Among these 29 genes, 3 represented one copy among triplicated genes, 11 were one copy of duplicated genes and 15 were single-copy genes in *B. rapa*. Of the 15 single-copy genes, 7 were not found either in *B. oleracea* or *A. thaliana*, indicating that those genes are unique to *B. rapa*, and BraTo-9 could have a role in the evolution of these unique MITE insertion-containing genes (Table 1). Comparative analysis of a BraTo-9-inserted gene (Bra016667) and its homologs from *B. rapa*, *B. oleracea* and *A. thaliana* showed that the structure of the Bra016667 gene was altered by the inclusion of one additional exon derived from the BraTo-9 MITE sequence (Figure 5a, b). Bra016667 was predicted to encode 162 amino acid residues, compared to 64 in its non-inserted homologs (Figure S3 in File S1).

**Table 1:** Comparison of 29 genes harboring BraTo-9 fragments with their homologous genes.

MITE ID^a^	Alignment Length	Ortholog from *A. thaliana*	Triplicated blocks in *B. rapa* b			Triplicated blocks in *B. oleracea* b			Function
			LF	MF1	MF2	LF	MF1	MF2	
4830	249	AT1G15270	Bra026150	Bra026774	**Bra016667**	Bol038124	Bol029240	Bol031556	Translation machinery associated-7
4696	83	AT1G29540	Bra032324	Bra010824	**Bra030143**	Bol022477	Bol008567	Bol041891	Paclobutrazol resistance 5
4791	104	AT4G00740	**Bra037356**	Bra000959	Bra008529	Bol011493	Bol010765	Bol040633	Quasimodo 3
4761	64	AT2G27900	-	Bra034375	**Bra000500**	-	Bol026404	-	Unknown protein
4840	174	AT5G58003	**Bra002681**	-	Bra006789	Bol012174	Bol026003	-	C-Terminal domain phosphatase-like 4
4685	124	AT1G32380	Bra023263	**Bra010159**	-	Bol022230	-	Bol020910	Hosphoribosyl pyrophosphate synthase 2
4702	174	AT3G55550	-	**Bra014780**	Bra003216	-	-	Bol034192	Concanavalin a-like lectin protein kinase family
4707	174	AT2G36480	Bra005265	**Bra017233**	-	Bol011739	Bol037712	-	Fructose-Bisphosphate Aldolase 6
4759	147	AT4G24840	Bra013840	**Bra019205**	-	Bol039469	Bol042194	-	Molecular function unknown
4675	109	AT1G11430	**Bra019853**	-	Bra016835	Bol036645	Bol001971	-	Multiple organellar RNA editing factor 9
4716	85	AT2G30440	-	**Bra021634**	Bra022799	-	Bol033345	-	Plastidic type I signal peptidase 2b
4739	85	AT3G14415	**Bra027338**	Bra021556	Bra001550	-	-	-	Glycolate oxidase 2
4781	174	AT3G28857	Bra025358	Bra033063	**Bra039043**	Bol042990	Bol033198	-	Paclobutrazol resistance 5
4847	156	AT5G44370	Bra033741	**Bra039510**	-	Bol006387	-	-	Phosphate transporter 4–6
4787	174	AT3G60660	**Bra007549**	-	-	Bol045621	-	-	Molecular function unknown
4652	223	AT4G00540	-	-	**Bra008522-**		**-**	Bol040626	C-Myb-like transcription factor 3r-2
4684	64	AT1G48580	**Bra018738**	-	-	Bol045135	-	-	Molecular function unknown
4674	175	AT2G02970	**Bra024796**	-	-	Bol007213	-	-	gda1/cd39 Nucleoside phosphatase family protein
4672	252	AT3G29130	**Bra025377**	-	-	Bol043009	-	-	Molecular function unknown
4851	174	AT4G27060	**Bra026382**	-	-	-	-	-	Convoluta, spiral 2, spr2, tor1, tortifolia 1
4872	161	AT2G15290	-	-	**Bra039835**	-	Bol019351	-	Attic21, Chloroplast import apparatus 5
4705	173	-		**Bra014846**	**-**	-	-	Bol034192	-
4748	174	-	-	-	**Bra001902**	-	-	-	-
4692	252	-	-	-	**Bra003971**	-	-	-	-
4786	174	-	**Bra007001**	**-**	-	-	-	-	-
4780	174	-	**Bra007484**	-	-	-	-	-	-
4752	127	-		**Bra012692**	**-**	-	-	-	-
4782	227	-	-	-	**Bra017374**	-	-	-	-
4703	174	-	-	**Bra035666**	-	-	-	-	-

a MITE position and alignment information can be found in Table S3.

b Triplicated paralogs of *B. rapa* and orthologs from *A. thaliana* and *B. oleracea* were identified from BRAD annotation information. Bold indicates the gene with BraTo-9 insertion.

### MIP analysis with *Brassica* relatives

**Figure 6:**
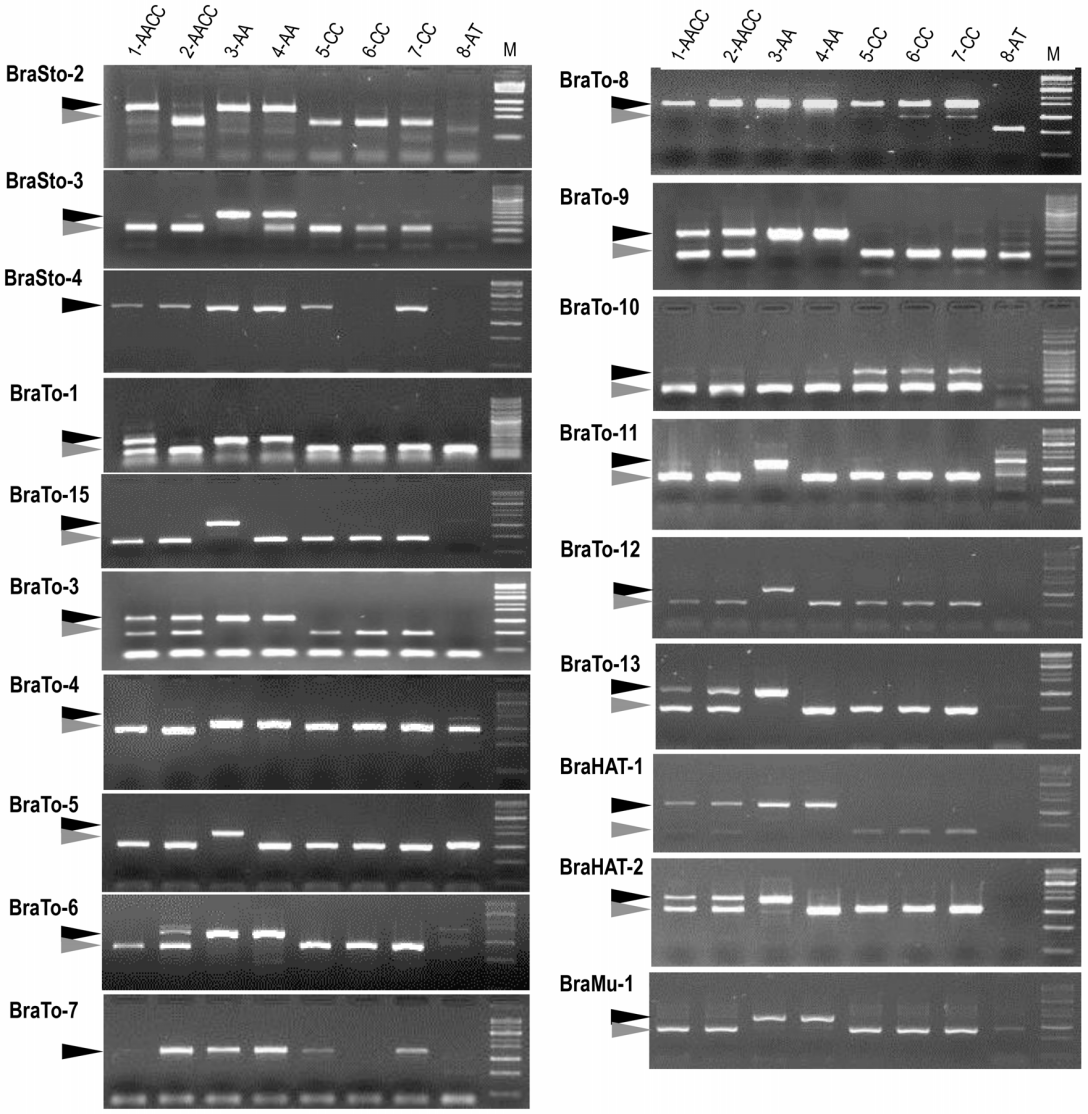
MITE insertion polymorphism (MIP) analysis of 19 MITE families in the *Brassica* genome. The accessions used here: 1- *B. napus* (Tapidor), 2- *B. napus* (Ningyou 7), 3- *B. rapa* (Chiifu), 4- *B. rapa* (Kenshin), 5- *B. oleracea* (C1234), 6- *B. oleracea* (C1184), 7- *B. oleracea* (C1235), 8- *A. thaliana* (Columbia). M, molecular size marker. *Black* and *gray* arrowheads indicate the products with and without MITE insertion, respectively.

To confirm MITE activity and insertion polymorphism, 346 primer pairs for 187 Br-members and 159 Bo-members belonging to the 19 MITE families were used to analyze against 3 different *Brassica* accessions. Among the 346 primers, 162 (87%) and 127 (80%) primer pairs derived from Br- and Bo-members, respectively, produced the expected amplification profile (Table 2: Tables S7 and S8 in File S1). A representative MIP profile for each family is shown in Figure 6. Out of 289 markers, 150 (52%) produced polymorphic patterns, and 66 (23%) of those revealed intra-species polymorphisms. Most of the MITE families showed polymorphism within or between species, suggesting that they have been active recently, whereas BraTo-7, 10 and BraHAT-1 members showed only interspecies-level MIP. BraSto-2, 3, BraTo-1, 2, 4, 5, 6, 8, 9, 11 and BraHAT-2 exhibited high rates of MIP (50%–80%) (Table 2; Tables S7 and S8 in File S1). By contrast, there was less than 50% MIP for BraSto-4 and BraTo-3, 7, 10, 12, 13 and BraMu-1. Overall insertion polymorphism analysis of the three different *Brassica* species revealed most of the MITE families are active and provide abundant valuable genomic resources in the *Brassica* genome.

**Table 2:** Summary of MITE insertion polymorphism analysis.

MITE family	No. of Primers analyzed^a^	No. of polymorphic markers				
		Intra-species level				Inter and Intra-species level
		*B. napus*	*B. rapa*	*B. oleracea*	Total	
BraSto-2	117	9	8	3	20 (17%)	61 (52%)
BraSto-3	5	0	2	0	2 (40%)	4 (80%)
BraSto-4	14	0	2	2	4 (29%)	4 (29%)
BraTo-1	22	1	2	4	7 (32%)	13 (59%)
BraTo-2	13	1	4	1	5 (38%)	8 (62%)
BraTo-3	8	0	2	0	2 (25%)	3 (38%)
BraTo-4	8	2	1	0	3 (38%)	5 (63%)
BraTo-5	11	0	3	1	4 (36%)	9 (82%)
BraTo-6	8	1	0	0	1 (13%)	5 (63%)
BraTo-7	9	0	0	0	0 (0%)	2 (22%)
BraTo-8	6	1	0	0	1 (17%)	3 (50%)
BraTo-9	23	3	4	3	9 (39%)	14 (61%)
BraTo-10	8	0	0	0	0 (0%)	2 (25%)
BraTo-11	4	2	1	0	2 (50%)	3 (75%)
BraTo-12	5	0	0	1	1 (20%)	2 (40%)
BraTo-13	7	0	1	0	1 (14%)	2 (29%)
BraHAT-1	7	0	0	0	0 (0%)	3 (43%)
BraHAT-2	7	1	2	0	3 (43%)	5 (71%)
BraMu-1	7	1	0	0	1 (14%)	2 (29%)
	289	22	32	15	66 (23%)	150 (52%)

a A total of 162 and 127 MIP primer pairs were analyzed based on the MITE members in genome sequences of *B. rapa* and *B. oleracea*, respectively. A detailed summary of MIP analysis for *B. rapa* and *B. oleracea* is given in Table S7 and S8, respectively.

## Discussion

### Characterization of 20 MITE families reveals MITE dynamics in the *Brassica* genome

We performed genome-wide systematic analysis to identify novel MITE families in the recently published genome sequences of *B. rapa* and *B. oleracea* using a computational tool, MITE-Hunter [38]. Overall, 18 MITE families were newly identified from 320 modeled MITEs after manual editing based on signature MITE structures. Recent genome-wide characterization of MITEs using three different *in silico* tools has revealed 174 MITE families in *B. rapa* reported in the P-MITE database [26]. Though our analysis using only a single tool, MITE-Hunter, we identified three new MITE families in *B. rapa* that were not present in the P-MITE database, one of which was homologous to a family reported in *A. thaliana* [26]. We chose to use the MITE-Hunter tool because of its efficiency in MITE detection and relatively low false positive rates compared to other tools. For example, only 17 MITE families were identified as genuine from the 1350 predicted structures using the MUST program in silkworm [57].

The 20 MITE families examined herein of the *Brassica* genomes, including two previously reported families, were classified into four superfamilies based on their TSDs (Figure 1; Table S2 in File S1). Four, two and one MITE family belonged to the *Stowaway*, *hAT* and *Mutator* superfamilies, respectively, whereas 14 belonged to the *Tourist* superfamily, which is one of the predominant superfamilies in *Brassica* [26]. Though *Tourist* MITEs are thought to have evolved as deletion derivatives from autonomous elements, we were not able identify their putative transposase partner. The presence of MITE-derived miRNAs suggests that MITEs might influence the regulation of gene expression and activation of related MITEs and TEs [33]–[36], [58]. Ten miRNA families of *A. thaliana* showed high similarity to 10 different MITE family sequences. We could not identify complete structures for nine of the ten MITE families in *A. thaliana*, suggesting that the nine family members were not activated but instead degenerated in *A. thaliana*. More in depth analysis is required to elucidate the biogenesis of MITE-derived miRNAs and the potential functional roles of such miRNAs.

### MITEs were actively amplified at gene-rich regions in *Brassica* genome

A total of 5894 Br-members and 6026 Bo-members belonging 20 MITE families were retrieved from *B. rapa* and *B. oleracea*, respectively. Only four families with 573 members have been identified in the *A. thaliana* genome, suggesting that MITE evolution, amplification and burst occurred in the *Brassica* genus after divergence with *Arabidopsis* 17 MYA [7], [9]. The total number of Br-and Bo-members were similar and the numbers of members of 13 families were also similar, suggesting that the major members of 13 MITE families evolved before the divergence of the two species 4 MYA. However, seven MITE families (BraSto-3, BraTo-1, 4, 7, 9, 11 and BraMu-1) displayed large variation in copy numbers between the two species. In particular, BraTo-1 was represented by 1216 members in the *B. oleracea* genome but only 207 copies in *B. rapa*, suggesting that these MITE families actively amplified after the divergence of *B. rapa* and *B. oleracea* around 4 MYA [45].

Members of the 20 MITE families are widely distributed throughout the pseudo-chromosome sequences of *B. rapa* and *B. oleracea* (Figure S2 in File S1). There is much evidence that MITEs are associated with gene and gene-rich regions [20], [30], [59]–[61], and MITEs mostly reside in genic regions such as promoters, 5′- and 3′-UTRs, introns and CDSs, which may influence the expression of genes by providing regulatory sequences or recruiting epigenetic modifications [27], [30], [62]. In the present study, 3120 out of 5761 (54%) Br-members were found within the 2-kb genic regions, which is a higher frequency than would be expected by random transposition. Additionally, 51% (3068/6026) of Bo-members were present in or near genic regions in the *B. oleracea* genome, suggesting that *Bo*-MITEs are even more closely associated with genes than are *Br*-MITEs.

### BraTo-9 could play a role in the evolution of duplicated genes in *B. rapa*

We found that especially BraTo-9 MITE family members preferentially reside in genic regions, potentially providing novel exons for functional genes (Table 1). To illustrate this possibility, we demonstrated that the structure of a *B. rapa* gene (Bra016667) was modified by BraTo-9 insertion (Figure 5). The non-inserted ortholog of Bra016667 from *A. thaliana* (AT1G15270, *TRANSLATION MACHINERY ASSOCIATED7*) has an important functional role in protein translation, and deletion of this gene results in alteration of the protein biosynthesis rate [63]. We found that when BraTo-9 insertion occurred in triplicated or duplicated genes in *B. rapa*, it was always present in only one of duplicated or triplicated genes, suggesting that the BraTo-9 members were actively amplified in *B. rapa* after divergence with *B. oleracea* 4 MYA [2], [7].

Unlike other MITEs, BraTo-9 members are rich in G+C (61%), which may be a crucial factor aiding their preferential incorporation into exonic regions. It is tempting to speculate that the high G+C content of BraTo-9 members and their preferential location in genic regions are due to acquisition and adaptation from coding sequences or transposases (Table 1). The G+C content of TEs has been suggested to be responsible for the high efficiency of TE excision and integration [64], [65].

### MITEs as valuable sources of DNA markers

The principle characteristics of MITEs, such as small size, genetically stability, high copy numbers, and close associations with genes, are useful for development of marker systems in plants and animals [41], [42], [44], [66]–[68]. Most DNA markers, like those based on simple sequence repeats (SSRs), amplified fragment length polymorphism (AFLP), random amplified polymorphic DNA (RAPD), and restriction fragment length polymorphism (RFLP), detect gradually and simultaneously accumulated mutations. Meanwhile the MIP markers detect InDel polymorphism derived from insertion in a certain genotype and lineage-based inheritance. MITE-based or MIP markers have been effectively utilized for genetic diversity, high density mapping, genomic and evolutionary studies in rice, wheat, soybean and *Brassica* [20], [44], [61], [69], [70]. DNA markers developed from a single *Tourist*-MITE, *mPing*, in rice detect >80% (150/183) polymorphism between two *japonica* rice lines, and have been effectively employed to map the QTL for heading date [69]. Abundant insertion polymorphism can be identified in a short period of time using the MITE display approach [21], [41], [42].

In our previous study, we developed markers from BraSto-2 and utilized them for diversity analysis of the *Brassica* population and identification of evolution dates of MITEs [20]. Here, MIP was surveyed using 289 MITE members and showed 52% (150) polymorphism between *Brassica* species, including 23% (66) at the intra-species level, suggesting that most MITEs are active in the *Brassica* genome. However, MITE families such as BraTo-7, 10 and BraHAT-1 did not produce any polymorphism, suggesting that these MITE families have been silenced for a long time. We could also find MITEs that were activated after the diversification between *B. rapa* and *B. oleracea* 4 MYA. Most of the analyzed families showed moderate to high levels of MIP (13%–100%), suggesting that these MITE families were recently activated and randomly distributed among cultivars. Overall, the MIP markers developed in this study revealed considerable polymorphism in the *Brassica* species, and these DNA markers can be utilized for various genomics applications such as assessment of genetic diversity, association mapping, genotyping and identification of novel functional genes evolving from MITE insertion.

We have incorporated the MITE member and marker information reported herein into a database, BrassicaTED (http://im-crop.snu.ac.kr/ted/index.php), to promote its effective utilization for further studies [71].

## Conclusion

Genome-wide analyses of the *B. rapa* genome identified 18 previously uncharacterized MITE families belonging to the *Stowaway*, *Tourist*, *hAT* and *Mutator* superfamilies. We conducted a comparative genome-wide survey of around 12000 MITE members belonging to 20 families in *B. rapa*, *B. oleracea* and *A. thaliana*. We found that 52% (150/289) of MITE members have remained active since genome triplication 17 MYA in the *Brassica* genus based on MIP analysis, suggesting that MITE members played a dynamic role in the evolution of the *Brassica* genome. Our findings promote our understanding of MITE dynamics in the evolution of highly duplicated plant genomes and facilitate development of a variety of markers for breeding in *Brassica* species.

## Supporting Information

File S1 **Figure S1.** Representation of predicted secondary structure and expected loop formation of 20 MITE families used in this study. **Figure S2. Distribution of nearly intact MITE members in** ***B. rapa*** **,** ***B. oleracea*** **and** ***A. thaliana*** **.** The physical position information for the 20 MITE families in (A) *B. rapa* and (B) *B. oleracea* pseudo-chromosome sequences are listed in Table S3 and S4. (C) The distribution of members of four homologous MITE families in the *A. thaliana* genome. **Figure S3.** Comparisons of the amino acid sequences encoded by the MITE-inserted gene Bra016667 and its paralogs Bra026774, Bra026150 and orthologs (At1g15270, *TRANSLATION MACHINERY ASSOCIATED7*) and Bol038124, Bol029240 and Bol031556. The sequence added by the BraTo-9 insertion is indicated and highlighted. **Table S1.** Primers and polymorphism profile from MITE insertion polymorphism analysis of 19 MITE families. **Table S2.** Characteristics of 20 MITE families and their copy numbers in *B. rapa*, *B. oleracea* and *A. thaliana*. **Table S3.** Physical positions and characterization of the members of 20 MITE families in the *B. rapa* genome. **Table S4.** Physical positions and characterization of the members of 20 MITE families in the *B. oleracea* genome. **Table S5.** miRNAs associated with MITE families. **Table S6.** Analysis of MITE distribution in various genomic locations based on gene annotation in the *B. rapa* and *B. oleracea* genomes. **Table S7.** MITE insertion polymorphism analysis with primers based on *B. rapa* MITE members. **Table S8.** MITE insertion polymorphism analysis with primers based on *B. oleracea* MITE members.
(ZIP)
