# Correction: Genome-Wide Comparative Analysis of 20 Miniature Inverted-Repeat Transposable Element Families in *Brassica rapa* and *B. oleracea*

**DOI:** 10.1371/journal.pone.0103757

**Published:** 2014-07-22

**Authors:** The PLOS ONE Staff

There are errors in Figure 4 and Figure 6.

In the legend of Figure 4, the legend box color should be blue instead of red for BraTo-3 (Br-rich) and should be red for BraSto-4 (Bo-rich). Please see the corrected Figure 4 here.

**Figure 4:**
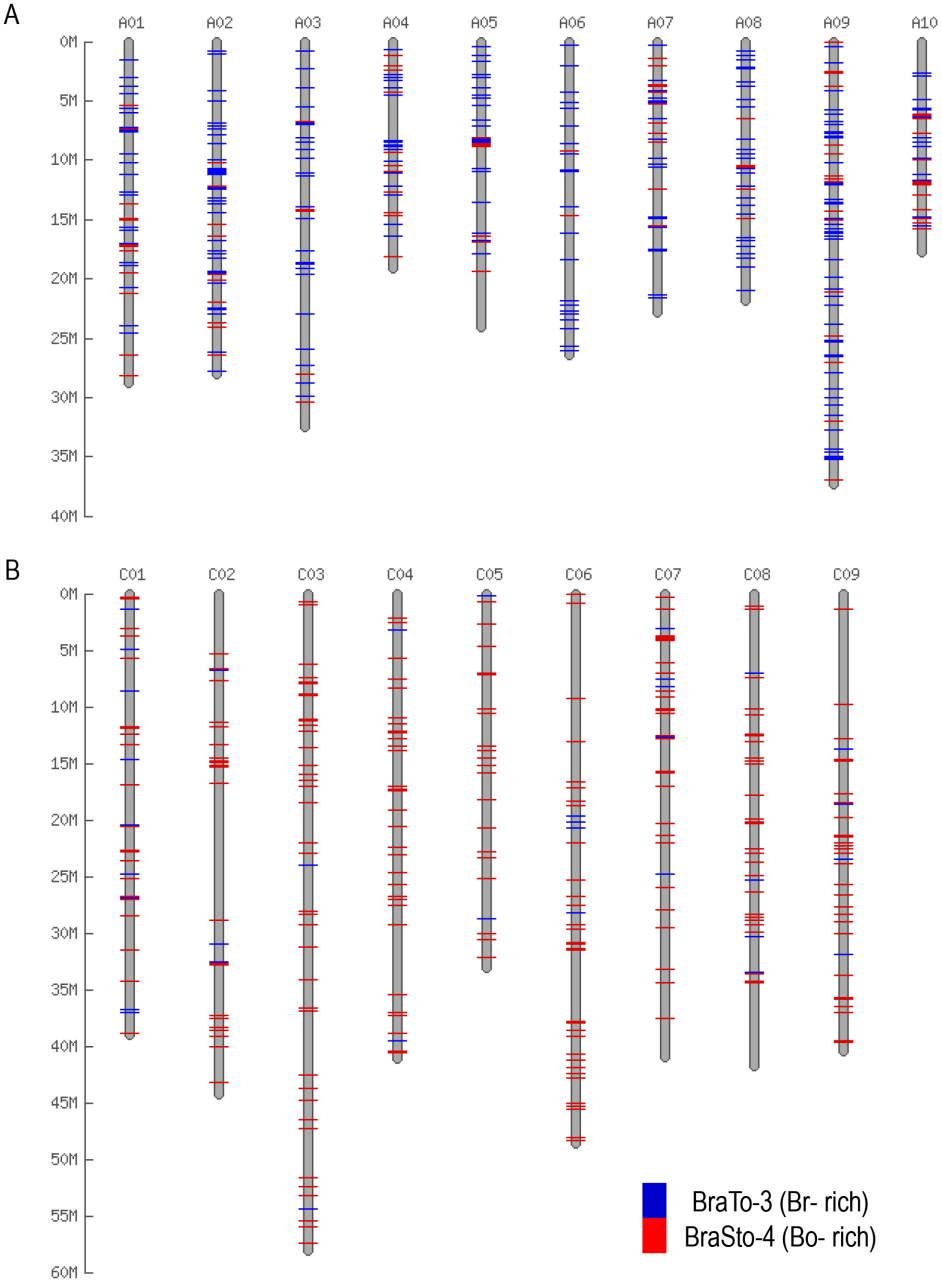
Differential distribution of MITE family members in *B. rapa* and *B. oleracea*. MITE families with intact members were used for *in silico* map construction on the 256 Mb *B. rapa* (A) and the 385 Mb *B. oleracea* (B) pseudo-chromosome sequences based on the physical positions. The physical position information for the MITE families of *B. rapa* and *B. oleracea* are listed in Table S3 and S4, respectively.

In the fifth blot of Figure 6 on the left, the caption BraTo-15 should be replaced with BraTo-2. Please see the corrected Figure 6 here.

**Figure 6:**
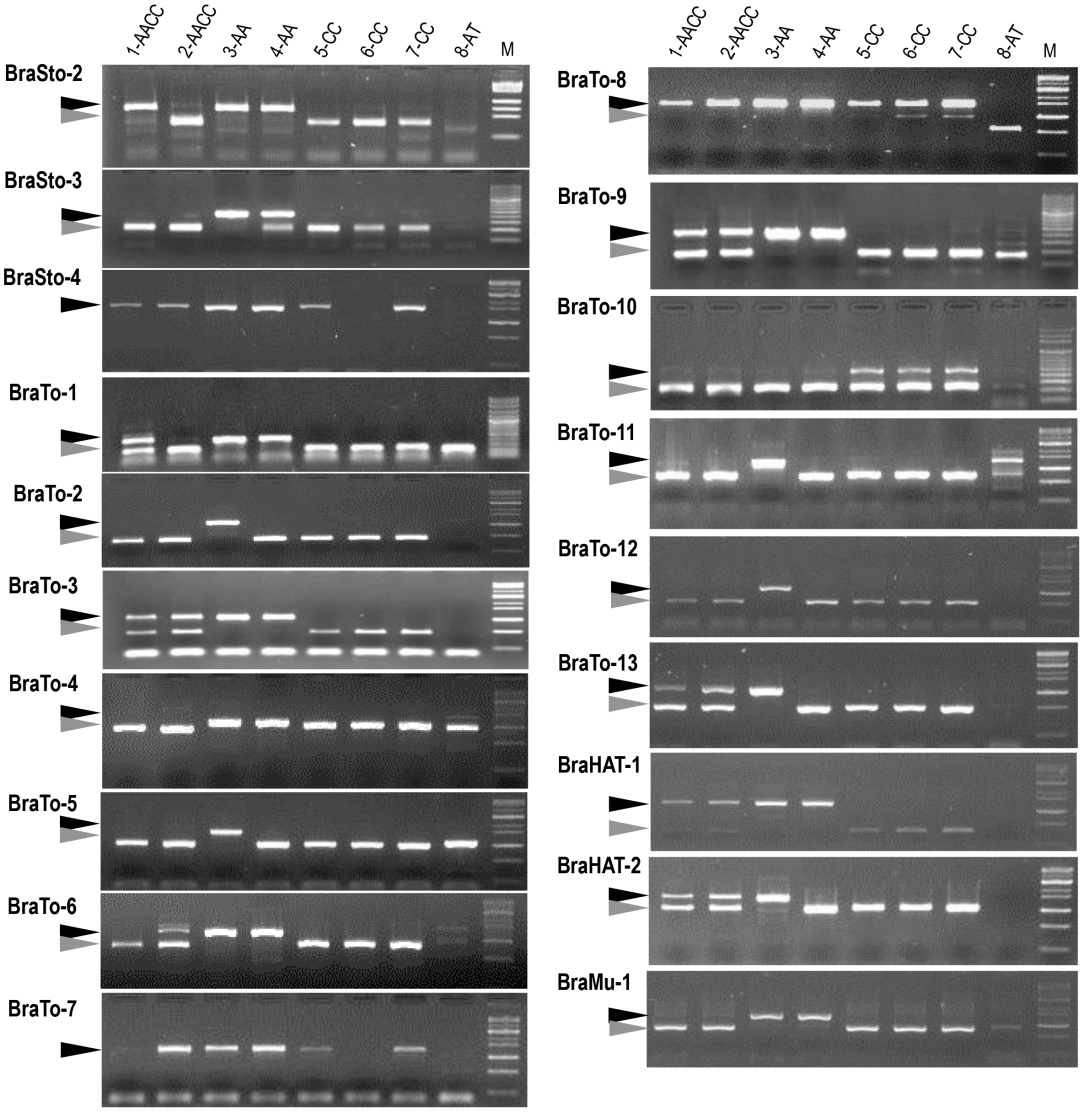
MITE insertion polymorphism (MIP) analysis of 19 MITE families in the *Brassica* genome. The accessions used here: 1- *B. napus* (Tapidor), 2- *B. napus* (Ningyou 7), 3- *B. rapa* (Chiifu), 4- *B. rapa* (Kenshin), 5- *B. oleracea* (C1234), 6- *B. oleracea* (C1184), 7- *B. oleracea* (C1235), 8- *A. thaliana* (Columbia). M, molecular size marker. *Black and gray* arrowheads indicate the products with and without MITE insertion, respectively.
