# Supplementary material for: Genome-Wide Comparative Analysis of 20 Miniature Inverted-Repeat Transposable Element Families in Brassica rapa and B. oleracea
Source: PLoS One. 2014 Apr 18;9(4):e94499. doi: 10.1371/journal.pone.0094499 (PMC3991616; doi:10.1371/journal.pone.0094499)
Supplement: File S1 — Figure S1. Representation of predicted secondary structure and expected loop formation of 20 MITE families used in this study. Figure S2. Distribution of nearly intact MITE members in B. rapa , B. oleracea and A. thaliana . The physical position information for the 20 MITE families in (A) B. rapa and (B) B. oleracea pseudo-chromosome sequences are listed in Table S3 and S4. (C) The distribution of members of four homologous MITE families in the A. thaliana genome. Figure S3. Comparisons of the amino acid sequences encoded by the MITE-inserted gene Bra016667 and its paralogs Bra026774, Bra026150 and orthologs (At1g15270, TRANSLATION MACHINERY ASSOCIATED7) and Bol038124, Bol029240 and Bol031556. The sequence added by the BraTo-9 insertion is indicated and highlighted. Table S1. Primers and polymorphism profile from MITE insertion polymorphism analysis of 19 MITE families. Table S2. Characteristics of 20 MITE families and their copy numbers in B. rapa, B. oleracea and A. thaliana. Table S3. Physical positions and characterization of the members of 20 MITE families in the B. rapa genome. Table S4. Physical positions and characterization of the members of 20 MITE families in the B. oleracea genome. Table S5. miRNAs associated with MITE families. Table S6. Analysis of MITE distribution in various genomic locations based on gene annotation in the B. rapa and B. oleracea genomes. Table S7. MITE insertion polymorphism analysis with primers based on B. rapa MITE members. Table S8. MITE insertion polymorphism analysis with primers based on B. oleracea MITE members. (ZIP) [file pone.0094499.s001.zip › MITE survey Supporting informations/Table S2 Characteristics of 20 MITE families and their copy numbers in B. rapa, B. oleracea and A. thaliana.docx]

**Table S2.** Characteristics of 20 MITE families and their copy numbers in *B. rapa, B. oleracea* and *A. thaliana*

| **MITE family** | **Superfamily** | **size (bp)** | | **TIR (bp)** | | **TSD (bp)** | | **A+T content (%)** | | ***Copies in B. rapa ^a^*** | | | | ***Copies in***  ***B. oleracea ^b^*** | | | | ***Copies in***   1. ***thaliana ^c^*** | | | | **Homologous element *^d^*** | | **Name in P-MITE DB *^e^*** | |  |
| --- | --- | --- | --- | --- | --- | --- | --- | --- | --- | --- | --- | --- | --- | --- | --- | --- | --- | --- | --- | --- | --- | --- | --- | --- | --- | --- |
|  |  |  |  |  |  |  |  |  |  | ***Total*** | | ***80:80 coverage*** | | ***Total*** | | ***80:80 coverage*** | | ***Total*** | | ***80:80 coverage*** | |  |  |  |  |  |
| BraSto-1 | *Stowaway* | | 267 | | 30 | | 2 | | 76 | | 267 | | 16 | | 250 | | 50 | | 0 | | 0 | | [[47](#_ENREF_47)] | | DTT_Brr1 | |
| BraSto-2 | *Stowaway* | 260 | | 34 | | 2 | | 74 | | 697 | | 401 | | 567 | | 210 | | 69 | | 35 | | [[20](#_ENREF_20)] | | DTT_Brr3 | |  |
| BraSto-3 | *Stowaway* | 242 | | 39 | | 2 | | 70 | | 14 | | 6 | | 5 | | 2 | | 0 | | 0 | | METMET | | DTT_Brr10 | |  |
| BraSto-4 | *Stowaway* | 558 | | 24 | | 2 | | 83 | | 1369 | | 97 | | 1459 | | 336 | | 384 | | 52 | | ATHPOGON1 | | - | |  |
| BraTo-1 | *Tourist* | 212 | | 58 | | 3 | | 68 | | 207 | | 8 | | 1216 | | 127 | | 0 | | 0 | | - | | DTH_Brr5 | |  |
| BraTo-2 | *Tourist* | 366 | | 31 | | 3 | | 70 | | 222 | | 61 | | 158 | | 60 | | 3 | | 0 | | HARB-1N1_Stu | | DTH_Brr27 | |  |
| BraTo-3 | *Tourist* | 252 | | 85 | | 3 | | 63 | | 513 | | 245 | | 284 | | 116 | | 0 | | 0 | | - | | DTH_Brr1 | |  |
| BraTo-4 | *Tourist* | 160 | | 16 | | 3 | | 51 | | 636 | | 287 | | 120 | | 36 | | 0 | | 0 | | - | | DTH_Brr3 | |  |
| BraTo-5 | *Tourist* | 286 | | 16 | | 3 | | 69 | | 207 | | 118 | | 107 | | 37 | | 0 | | 0 | | - | | DTH_Brr15 | |  |
| BraTo-6 | *Tourist* | 257 | | 18 | | 3 | | 64 | | 167 | | 60 | | 219 | | 76 | | 0 | | 0 | | - | | DTH_Brr22 | |  |
| BraTo-7 | *Tourist* | 366 | | 36 | | 3 | | 70 | | 213 | | 54 | | 460 | | 199 | | 85 | | 17 | | ATTIRX1B | | DTH_Brr17 | |  |
| BraTo-8 | *Tourist* | 348 | | 16 | | 3 | | 77 | | 137 | | 29 | | 117 | | 26 | | 32 | | 18 | | ATTIRX1C | | DTH_Brr19 | |  |
| BraTo-9 | *Tourist* | 264 | | 46 | | 3 | | 39 | | 227 | | 20 | | 103 | | 32 | | 0 | | 0 | | - | | DTH_Brr14 | |  |
| BraTo-10 | *Tourist* | 255 | | 18 | | 3 | | 69 | | 138 | | 35 | | 191 | | 50 | | 0 | | 0 | | - | | DTH_Brr20 | |  |
| BraTo-11 | *Tourist* | 305 | | 45 | | 3 | | 57 | | 4 | | 4 | | 16 | | 5 | | 0 | | 0 | | - | | - | |  |
| BraTo-12 | *Tourist* | 273 | | 46 | | 3 | | 60 | | 239 | | 66 | | 180 | | 67 | | 0 | | 0 | | - | | DTH_Brr8 | |  |
| BraTo-13 | *Tourist* | 268 | | 24 | | 3 | | 63 | | 140 | | 74 | | 174 | | 85 | | 0 | | 0 | | - | | DTH_Brr21 | |  |
| BraHAT-1 | *hAT* | 439 | | 35 | | 8 | | 67 | | 339 | | 24 | | 318 | | 55 | | 0 | | 0 | | - | | DTA_Brr55 | |  |
| BraHAT-2 | *hAT* | 248 | | 17 | | 8 | | 58 | | 17 | | 16 | | 22 | | 19 | | 0 | | 0 | | - | | DTA_Brr53 | |  |
| BraMu-1 | *Mutator* | 271 | | 56 | | 10 | | 74 | | 141 | | 24 | | 60 | | 16 | | 0 | | 0 | | - | | - | |  |
| Total |  |  | |  | |  | |  | | 5894 | | 1645 | | 6026 | | 1604 | | 573 | | 122 | |  | |  | |  |

*^a^* MITE copy and MITE fraction analysis were done using the available 283 Mb whole-genome pseudo-chromosome sequences of *B. rapa*

*^b^*MITE copies were analyzed from the available 385 Mb whole-genome pseudo-chromosome sequences of *B. oleracea*

*^c^*MITE copies were analyzed from 119 Mb (TAIR 10 version) whole-genome chromosome sequences of *A. thaliana*

*^d^* Homology-based search against the Repbase repeat database (<http://www.girinst.org/>)

*^e^*Homologous element found in the P-MITE database
